# Immune Responses in Pigs Vaccinated with Adjuvanted and Non-Adjuvanted A(H1N1)pdm/09 Influenza Vaccines Used in Human Immunization Programmes

**DOI:** 10.1371/journal.pone.0032400

**Published:** 2012-03-09

**Authors:** Eric A. Lefevre, B. Veronica Carr, Charlotte F. Inman, Helen Prentice, Ian H. Brown, Sharon M. Brookes, Fanny Garcon, Michelle L. Hill, Munir Iqbal, Ruth A. Elderfield, Wendy S. Barclay, Simon Gubbins, Mick Bailey, Bryan Charleston, COSI

**Affiliations:** 1 Institute for Animal Health, Compton near Newbury, United Kingdom; 2 Division of Veterinary Pathology, School of Clinical Veterinary Science, University of Bristol, Langford, Bristol, United Kingdom; 3 Animal Health and Veterinary Laboratories Agency - Weybridge, EU/OIE/FAO Reference Laboratory for Avian Influenza and Newcastle Disease, Addlestone, United Kingdom; 4 Department of Virology, Imperial College London, London, United Kingdom; 5 Institute for Animal Health, Pirbright, United Kingdom; The University of Hong Kong, China

## Abstract

Following the emergence and global spread of a novel H1N1 influenza virus in 2009, two A(H1N1)pdm/09 influenza vaccines produced from the A/California/07/09 H1N1 strain were selected and used for the national immunisation programme in the United Kingdom: an adjuvanted split virion vaccine and a non-adjuvanted whole virion vaccine. In this study, we assessed the immune responses generated in inbred large white pigs (Babraham line) following vaccination with these vaccines and after challenge with A(H1N1)pdm/09 virus three months post-vaccination. Both vaccines elicited strong antibody responses, which included high levels of influenza-specific IgG1 and haemagglutination inhibition titres to H1 virus. Immunisation with the adjuvanted split vaccine induced significantly higher interferon gamma production, increased frequency of interferon gamma-producing cells and proliferation of CD4^−^CD8^+^ (cytotoxic) and CD4^+^CD8^+^ (helper) T cells, after *in vitro* re-stimulation. Despite significant differences in the magnitude and breadth of immune responses in the two vaccinated and mock treated groups, similar quantities of viral RNA were detected from the nasal cavity in all pigs after live virus challenge. The present study provides support for the use of the pig as a valid experimental model for influenza infections in humans, including the assessment of protective efficacy of therapeutic interventions.

## Introduction

In June 2009, the World Health Organization (WHO) declared an H1N1 influenza pandemic in response to the emergence and global spread of a novel H1N1 influenza A virus - A(H1N1)pdm/09 (http://www.who.int/mediacentre/news/statements/2009/h1n1_pandemic_phase6_20090611/en/index.html), which contained a unique combination of gene segments derived from multiple viruses that have been circulating in pigs for decades [1]. Most A(H1N1)pdm/09 cases in humans resulted in mild illnesses, but in some people, more serious symptoms and fatalities have been reported [2].

In the UK, two A(H1N1)pdm/09 influenza vaccines produced from the A/California/07/09 H1N1 strain (Cal07) were used for the national immunisation programme: (i) Pandemrix (GlaxoSmithKline Biologicals S.A., GSK), an AS03b-adjuvanted split virion vaccine derived from embryonated chicken eggs, administered once in healthy adults, and (ii) Celvapan (Baxter AG), a non-adjuvanted whole virion vaccine derived from Vero cell culture, administered twice with a minimum of 3 weeks between injections. These A(H1N1)pdm/09 vaccines were authorized under “Exceptional Circumstances” on the basis of limited to very limited safety and immunogenicity data obtained with these A(H1N1)pdm/09 influenza vaccines and also utilised more complete safety and immunogenicity data obtained with H5N1 “mock-up” vaccines - e.g. similar versions of the A(H1N1)pdm/09 vaccines that contain the whole H5N1 A/Vietnam/1203/2004 influenza virus for the non-adjuvanted whole vaccine or the viral surface protein haemagglutinin (HA) derived from H5N1 A/Vietnam/1194/2004 for the adjuvanted split vaccine (product characteristics described in http://www.ema.europa.eu/docs/en_GB/document_library/Other/2010/01/WC500059182.pdf and http://www.dossiers-sos-justice.com/media/00/01/1744671157.pdf, respectively). For both vaccines, the immunogenicity data at the time of authorization was based on the generation of anti-HA antibodies following vaccination with either the A(H1N1)pdm/09 and/or “mock-up” vaccines, and included some antigenic cross-reactivity data. For the adjuvanted split vaccine, non-clinical studies were solely based on results obtained following the vaccination and challenge of ferrets with the “mock-up” vaccine. For the non-adjuvanted whole vaccine, two non-clinical studies were performed in ferrets vaccinated with the “mock-up” vaccine and one study was performed in mice using the A(H1N1)pdm/09 vaccine. Regarding the latter study, Kistner et al. recently reported that the non-adjuvanted whole vaccine provided protection against challenge with Cal07 with respect to undetectable virus titers in the lung tissue of vaccinated CD1 mice [3]. Since these A(H1N1)pdm/09 vaccines were made available, several authors have reported that both were strongly immunogenic in adults and/or children [4], [5].

The generation of neutralising antibodies against antigenic sites on the HA glycoprotein of the influenza virus (as assessed by HA inhibition assay or microneutralisation assay) is regarded as the criterion for evaluating immunity to influenza viruses and is believed to constitute the main correlate of protection [5], [6], [7]. Although cell-mediated immunity also correlates with the rate of viral clearance and protection of the respiratory tract after challenge with infectious influenza viruses [8], cellular-mediated immune responses have not been assessed following vaccination with the A(H1N1)pdm/09 vaccines and only limited data is available on the cellular-mediated immune responses elicited by influenza vaccines in general [9].

Mice and ferrets are the most routinely used animal models for the study of influenza infections and in particular for the evaluation of human influenza vaccines [10], [11]. In contrast, the pig is not currently considered as a major experimental model for influenza viruses, despite the fact that influenza viruses are enzootic in pigs [12] and several studies show the pig as a valuable model to study human influenza viruses: human influenza viruses do replicate to a similar level in both the upper and lower respiratory tract explants of pigs, which exhibit a similar sialic acid receptor distribution and pattern of virus attachment to humans [13], [14], and infections of pigs with human influenza viruses under natural conditions occur regularly [12]. Moreover, several studies have now demonstrated in pigs the pathogenesis and transmission of A(H1N1)pdm/09 influenza virus that emerged in 2009 [15], [16], [17]. In the present study, we assessed the immune responses generated to intramuscular immunisation with two commercial human influenza vaccines and challenge in inbred large white pigs (Babraham line).

## Materials and Methods

### Ethics Statement

All experiments were approved by the ethical review processes at the Institute for Animal Health (IAH) and Animal Health and Veterinary Laboratories Agency (AHVLA), were in accordance with national guidelines on animal use, and all efforts were made to minimise suffering of animals.

### Vaccines

Two commercial A(H1N1)pdm/09 influenza vaccines produced from Cal07 were used in this study: an AS03b-adjuvanted split virion vaccine (Pandemrix, GlaxoSmithKline Biologicals S.A., GSK, Rixensart, Belgium) and a non-adjuvanted whole virion vaccine (Celvapan, Baxter AG, Vienna, Austria).

The adjuvanted split virion vaccine was manufactured from a reassortant strain combining the HA, neuraminidase (NA) and viral polymerase subunit (PB1) genes of Cal07 and the PR8 strain backbone, generated by classical reassortment in embryonated chicken eggs. This vaccine was supplied as a suspension (antigen) and emulsion (adjuvant) multidose vial, and the volume after mixing one vial of suspension (2.5 ml) with one vial of emulsion (2.5 ml) corresponded to ten doses of vaccine. One dose (0.5 ml) contained: (i) 3.75 µg HA antigen; (ii) the oil-in-water AS03 adjuvant composed of squalene (10.69 mg), DL-α-tocopherol (11.86 mg) and polysorbate 80 (4.86 mg); and (iii) the excipient thiomersal (5 mg). The vaccine vials were stored at 2–8°C and used within 24 hours following mixing.

The non-adjuvanted whole virion vaccine was manufactured from Cal07, grown in Vero cells and inactivated (formaldehyde and UV irradiation). This vaccine was supplied as a 5 ml multidose vial, corresponding to ten vaccine doses. One dose (0.5 ml) contained 7.5 µg HA antigen. The vaccine vials were stored at 2–8°C and used within 3 hours following opening of the vial.

### Study design

Inbred large white pigs (Babraham line) were conventionally reared at the Institute for Animal Health (Compton, UK), with ages ranging from three to four months and weighing 15–29 kg at the initiation of the study. Prior to the study, all pigs were bled and shown by ELISA to be negative for anti-influenza A virus antibodies in the serum (i.e. not previously exposed to influenza A viruses). The pigs (three animals per group) were intramuscularly injected in the neck with one of the two commercial A(H1N1)pdm/09 influenza vaccines mentioned above (0.5 ml per pig). The vaccination regime was similar to that recommended for adult humans: the adjuvanted split virion vaccine was administered only once, whereas the whole virion vaccine was administered twice (two doses three weeks apart). Another two pigs were mock-vaccinated, i.e. received a single intramuscular injection in the neck with 2.5 µl uninfected allantoic fluid (corresponding to approximately 7.5 µg total egg protein) diluted in a mixture of 0.25 ml PBS and 0.25 ml adjuvant (emulsion vial from the adjuvanted split virion vaccine) per pig. Blood samples were collected by anterior vena cava venepuncture at various time-points following vaccination.

Three months after the initial vaccination, all pigs were intranasally inoculated with 10^6^ pfu of infectious A/England/195/09 H1N1 (Eng195 - 10^6^ pfu/4 ml – 2 ml per nostril), using a mucosal atomisation device (MAD® Nasal, Wolfe Tory Medical Inc., Salt Lake City, UT) to mimic aerogenous infection that results in the infection of both the upper and lower respiratory tract [16]. This infectious virus strain was propagated in MDCK cells. Clinical parameters (demeanour, respiratory signs, faeces consistency and rectal temperature) were assessed and nasal swab samples were collected one day before and every day after challenge for 9 days. The swabs were stored in 1 ml RNAlater (Applied Biosystems/Ambion, Austin, TX) and stored frozen at −80°C until tested by a modified influenza A M gene real-time RT-PCR assay (RRT-PCR, as described below).

### Culture medium

The culture medium was RPMI-1640 medium with glutamax-I and 25 mM Hepes (Invitrogen Ltd., Paisley, UK) supplemented with penicillin (100units/ml), streptomycin (100 µg/ml), 1× non-essential amino acids, 1 mM sodium pyruvate and 10% heat-inactivated pig serum (Invitrogen Ltd.), and was subsequently termed complete medium. The commercial pig serum used in the culture medium was checked to be negative for antibodies against four swine influenza viruses mostly endemic in UK pigs (avian-like H1N1[195852], classical H1N1, H1N2 and H3N2) and the pandemic Eng195 (data not shown).

### Isolation and stimulation of pig peripheral blood mononuclear cells (PBMC)

Blood (25–40 ml per pig) was collected in heparinised tubes at various time-points post vaccination (i.e. day 0, 7, 12, 21, 28, 33, 93 and 100), and PBMC were obtained by density gradient centrifugation (1200×g for 30 min over Histopaque® 1.083 g/ml, Sigma-Aldrich, Poole, UK). 2×10^5^ PBMC per well were cultured (10 replicates per condition) in U-shape 96-well microtiter plates in 200 µl complete medium alone or with the following stimuli: 10^5^EID_50_/ml LBP-inactivated Cal07 (grown in embryonated chicken eggs, corresponding to a 1/1000 dilution of virus stock), 10^5^ pfu/ml UV-inactivated Eng195 (grown in MDCK cells, corresponding to a 1/20 dilution of virus stock), mock antigen (uninfected allantoic fluid diluted at 1/1000 or MDCK lysate diluted at 1/20) or 1 µg/ml pokeweed mitogen (PWM, Sigma-Aldrich). PBMCs were then incubated for 3 days at 37°C in a 5% CO_2_ incubator, before harvesting cell supernatants to assess interferon gamma (IFNγ) production by ELISA or harvesting cells to quantify IFNγ-producing cells by ELISPOT.

For the assessment of cell proliferation, a number of PBMC were labelled with carboxyfluorescein diacetate succinimydyl ester (CFSE) cell tracer (Invitrogen Ltd) immediately after their isolation. Briefly, PBMC (5×10^6^/ml in pre-warmed PBS) were incubated with 0.5 µg/ml CFSE in a water bath for 15 min at 37°C. Cells were recovered by centrifugation, suspended at 5×10^6^cells/ml in pre-warmed complete medium and incubated in a water bath for a further 30 min at 37°C. Subsequently, cells were washed twice, suspended at the appropriate concentration (2×10^6^cells/ml) in complete medium and stimulated for five days as described above for unlabelled PBMC.

### Swine IFNγ ELISA

Swine IFNγ was measured in cell supernatants of PBMC cultured for 3 days (as described above) by using a commercial ELISA kit (Thermo Scientific, Rockford, IL), according to the manufacturer's instructions. Concentrations of swine IFNγ in test samples were determined by extrapolation to the linear part of the standard curve and taking into account any extra dilution factor applied to the samples. Results were expressed as pg swine IFNγ/ml and the lower detection limit for the assay was 2 pg/ml.

### Swine IFNγ ELISPOT

MultiScreen™-HA ELISPOT plates (Millipore, Watford, UK) were coated for two hours at room temperature with 1 µg/ml mouse anti-pig IFNγ (BD Biosciences, San Jose, CA) in carbonate buffer (0.1 M Na_2_CO_3_/NaHCO_3_, pH 9.6). Plates were washed five times with PBS and subsequently incubated for two hours at 37°C with 100 µl per well of blocking buffer (PBS containing 4% dried skimmed milk). Plates were then washed five times with PBS and stored at 4°C until use.

For each culture condition, ten wells of 3 day-stimulated PBMC (as described above) were pooled and resuspended in 200 µl complete media (equivalent to 10^7^ initial cells/ml) and 2-fold serial dilutions were performed in complete medium down to 3.1×10^5^ cells/ml. 100 µl/well of each cell suspension was added to the coated ELISPOT plates and cultured overnight at 37°C in a 5% CO_2_ incubator. Cells were washed off the plates using PBS containing 0.05% Tween20, then 100 µl per well biotinylated mouse anti-pig IFNγ (0.5 µg/ml, BD Biosciences) was added to the plates for 2 hours at room temperature. Plates were washed five times with PBS containing 0.05% Tween20 and 100 µl per well streptavidin conjugated to alkaline phosphatase (1/1000, Invitrogen Ltd.) was added to the plates for 1 hour at room temperature. Plates were washed five times with PBS containing 0.05% Tween20 and 100 µl per well alkaline phosphatase substrate solution (Biorad laboratories, Hercules, CA) was added for 20 min at room temperature. Plates were then rinsed with tap water and allowed to dry overnight at room temperature before counting the dark blue-coloured immunospots using the AID ELISPOT reader (AID Autoimmun Diagnostika GmbH, Strassberg, Germany). Results were expressed as IFNγ-producing cell number per 10^6^ stimulated PBMC.

### Assessment of cell proliferation in T cell subsets

For each culture condition, ten wells of 5 day-stimulated CFSE-labelled PBMC (equivalent to 2×10^6^ initial cells) were pooled, resuspended in 100 µl FACS buffer (PBS containing 1% bovine serum albumin and 0.1% sodium azide) and analyzed by immunofluorescence using the following reagents: biotinylated mouse anti-porcine CD4 (clone MIL17, 1/300 of a monoclonal antibody purified from hybridoma culture supernatant and biotinylated at the IAH) and PE-conjugated mouse anti-porcine CD8α (clone 76-2-11, BD Biosciences) monoclonal antibodies, followed by incubation with APC-conjugated streptavidin (1/200, Southern Biotechnology Associates, Birmingham, AL). 50,000 viable cells per sample were analyzed using a FACScan flow cytometer (BD Biosciences).

### Detection of influenza-specific antibodies by ELISA

Serum was collected at various time-points post vaccination (i.e. day 0, 7, 12, 21, 28, 33, 93 and 100) for the assessment of influenza-specific antibodies by ELISA. 96-well ELISA plates were coated overnight at 4°C with pre-optimized concentrations of Eng195 grown in embryonated chicken eggs (1 mg/ml). Threefold dilutions of test sera and reference serum starting from 1/3 in PBS-Tween plus egg powder (1 mg/ml, to competitively inhibit any anti-egg antibody) were applied. Binding of antibodies was detected with monoclonal antibodies specific for porcine IgG1 (K139.3C8, AbD Serotec, Kidlington, UK) and IgG2 (K68.1G2, AbD Serotec), followed by goat anti-mouse IgG (Fc-specific) conjugated to horseradish peroxidase (Sigma-Aldrich), all used at optimal dilutions. Finally, ortho-phenylenediamine (OPD, Sigma-Aldrich) in carbonate buffer was added and the optical density values were read for each well at dual wavelengths (405 nm and 492 nm) using a Labsystems Multiskan plate reader (Fisher Scientific, Loughborough, UK). The reference serum (obtained at day 14 post-infection from a pig experimentally challenged with 10^6^EID_50_ of infectious Eng195) was assigned arbitrary antibody units (log 3) and serial dilutions were used to construct a standard curve. The quantities of antibody in test sera were determined by the interpolation of optical density values from all dilutions falling within the reference range. All antibody values were expressed relative to the standard as Log ELISA units (EU).

### Detection of haemagglutination inhibition (HI) titres

Antibody titres were measured by use of a HI assay following the protocol detailed thereafter. In brief, 100 µl serum samples were pre-treated with 400 µl receptor destroying enzyme (RDE, 1/20 dilution in calcium saline solution, Sigma-Aldrich) at 37°C overnight. 300 µl sodium citrate (3%) was added per sample and incubated for a further 30 minutes at 56°C for enzyme inactivation. The strain of influenza virus (Eng195) used in this assay was grown in embryonated chicken eggs and inactivated by treatment with beta-propiolactone. 25 µl (4 haemagglutination units) of influenza virus was incubated at 37°C for one hour with an equal volume of 2-fold serial dilutions of RDE-treated serum starting from 1/8 in V-shape 96-well microtiter plates. 50 µl of 1% (vol/vol in PBS) chicken red blood cells was then added to each well and incubated for 45 min at room temperature after gentle mixing. Haemagglutination inhibition was read immediately after the last incubation, and HI titers were expressed as the reciprocal of the highest dilution of serum where haemagglutination was prevented.

### Matrix (M) gene RRT-PCR

Total RNA was extracted with the RNeasy® mini kit (Qiagen, Crawley, UK) according to the manufacturer's instructions with minor modifications as described thereafter. Briefly, nasal swabs were thawed, vortexed and 600 µl of the swab storage medium (RNAlater) was clarified by centrifugation at 12,000×g for 10 min. 500 µl of the clarified supernatant was mixed with 1.5 ml RLT buffer (containing ß-mercaptoethanol) and 2 ml 75% ethanol, and applied to the RNeasy spin column. Subsequently, the kit protocol for purification of total RNA from animal cells was followed and total RNA was eluted in 30 µl RNase-free water.

The modified M gene RRT-PCR utilised the forward primer and probe originally described by Spackman et al [18], with a reverse primer modified to provide a perfect sequence match with the novel pandemic H1N1 virus, typified by Cal07 (accession number: FJ966975) [16]. The four altered nucleotides in the modified reverse primer are indicated in upper case: 5′-tgc aaa Gac aCT ttc Cag tct ctg-3′. The superscript III platinum one-step quantitative RT-PCR kit (Invitrogen Ltd.) was used with a 25 µl reaction mixture containing 0.5 µl of kit-supplied enzyme mixture (including RT and hot-start *Taq* polymerase), 0.9 µM of each primer, 0.4 µM probe, 12.5 µl 2× reaction mix (including MgSO_4_ and dNTPs), 0.5 µl of 25 nM ROX reference dye and 3 µl total RNA. RRT-PCR was performed with the 7500 Fast Real-Time PCR system thermocycler and sequence detection software (Applied Biosystems, Foster City, CA) as follows: (i) the RT step conditions were 5 min at 50°C and 2 min at 95°C; and (ii) the two-step PCR cycling protocol was 40 cycles of 95°C for 3 sec and 60°C for 30 sec. Fluorescence data were acquired at the end of each annealing step.

RNA from 500 µl RNAlater containing ten-fold dilutions of a titrated sample of MDCK-grown Eng195 (10^6^ to 10^1^ pfu) was extracted in a similar manner as RNA from nasal swabs. This served to calibrate the Ct values derived from testing extracted clinical specimens by the modified M gene RRT-PCR with an equivalent viral infectivity titre that is expressed in relative equivalent units (REU) [19].

### Statistical analysis

Time-course data (IFNγ ELISA/ELISPOT, IgG1/IgG2/HI titres, virus shedding and body temperature) were analysed using linear mixed models with animal as a random effect and an autoregressive, AR(1), correlation structure [20]. Where appropriate, data were log transformed. Model selection proceeded by stepwise deletion of non-significant terms (as judged by changes in the Akaike information criterion), starting from an initial model including day and vaccination as fixed effects and an interaction between these factors. However, exploratory analyses indicated substantial departures from normality when using linear mixed models for both IFNγ ELISA and HI titres, even after transformation. Accordingly, these data were analysed using Kruskal-Wallis tests to investigate the impact of vaccination at individual time-points.

Proliferation data for each T cell subset (CD4^+^CD8^−^, CD4^+^CD8^+^ or CD4^−^CD8^+^) were analysed using linear mixed models. Model selection proceeded by stepwise deletion of non-significant terms (as judged by changes in the Akaike information criterion), starting from an initial model including treatment and vaccination as fixed effects and an interaction between these factors and animal as a random effect.

As a measure of the total amount of viral RNA detected, the area-under-the-curve (AUC) was computed for each animal by applying the trapezium rule to the viral RNA data. AUC was compared between animals using a Kruskal-Wallis test.

All statistical modelling was carried out using the nlme package in R (http://www.R-project.org/). P values equal to or below 0.05 were considered statistically significant.

## Results

### High Ab titres were generated following vaccination with both the non-adjuvanted whole or adjuvanted split A(H1N1)pdm/09 influenza vaccines

**Figure 1:**
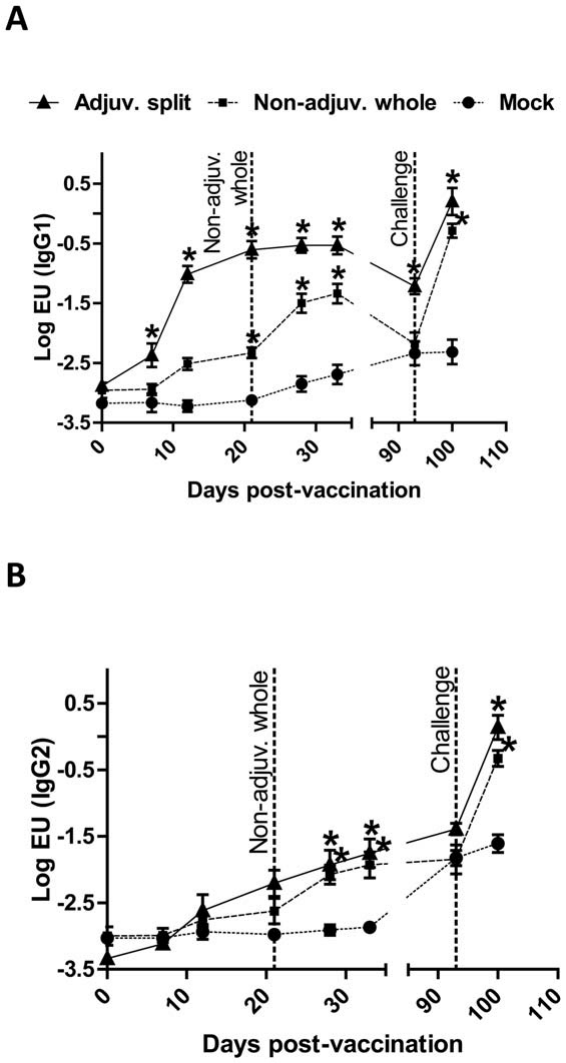
Antibody titre after vaccination and challenge. Pigs were vaccinated with the adjuvanted split (solide line), non-adjuvanted whole (dashed line) or mock (dotted line) vaccine. The non-adjuvanted whole vaccine was administered twice (at day 0 and day 21pv). At day 93pv, all pigs were challenged with Eng195. Serum samples were taken at various time-points post-vaccination and challenge, and IgG1 (**A**) and IgG2 (**B**) Ab titres were quantified by ELISA. Results are expressed as the mean Log EU value ± SEM. *, P<0.05.

Influenza-specific IgG1 and IgG2 Ab titres were quantified by ELISA using serum samples collected from each pig at various time points post-vaccination (pv). Both vaccines induced a significant systemic Ab response, which was predominantly of the IgG1 isotype (**Figure 1A and 1B**). Indeed, IgG1 titres were increased from −2.88±0.04LogEU at day 0pv to −0.53±0.15LogEU at day 33pv in pigs vaccinated with the adjuvanted split vaccine, whereas IgG2 titres only rose from −3.34±0.03LogEU at day 0pv to −1.75±0.21LogEU at day 33pv (n = 3). Similar results were obtained in pigs vaccinated with the non-adjuvanted whole vaccine. Moreover, pigs vaccinated with the adjuvanted split vaccine produced significantly more specific IgG1 antibodies after one vaccination, compared to those vaccinated with the non-adjuvanted whole vaccine (−0.61±0.14LogEU versus −2.33±0.09LogEU at day 21pv, respectively, P<0.05, n = 3). IgG1 Ab titres in pigs vaccinated with the non-adjuvanted whole vaccine were however boosted after the second vaccination, and finally reached high titres by day 33pv (−1.34±0.16LogEU, n = 3). Nevertheless, IgG1 titres were significantly higher for animals vaccinated with the adjuvanted split vaccine compared to those vaccinated with the non-adjuvanted whole vaccine from day 12pv to day 93pv (P<0.05, n = 3). Noticeably, both IgG1 and IgG2 Ab titres were significantly increased following intranasal challenge with infectious Eng195 influenza virus in all pigs vaccinated with the A(H1N1)pdm/09 influenza vaccines. At day 100pv (i.e. day 7 post-challenge), IgG1 and IgG2 Ab titres respectively reached −0.29±0.11LogEU and −0.33±0.12LogEU for pigs vaccinated with the non-adjuvanted whole vaccine, and 0.20±0.23LogEU and 0.14±0.18LogEU for pigs vaccinated with the adjuvanted split vaccine (n = 3). In contrast, influenza-specific IgG1 and IgG2 Ab titres were not increased in mock-vaccinated pigs at day 100pv, and it is likely this time-point was too early for their detection as high levels of Ab would only be expected from day 12–14 post-challenge in these naïve animals.

Since HI titres are considered correlates of protection against a subsequent challenge with infectious influenza virus, we also determined HI titres in the serum samples collected from each pig during the course of our study (**Table 1**). Similarly to the IgG1 titres, HI titres were significantly increased in pigs vaccinated with the adjuvanted split vaccine after only one injection (reaching 107±21 at day 28pv, P = 0.04, n = 3). Although undetectable in pigs vaccinated with the non-adjuvanted whole vaccine after the first injection, HI titres were also significantly increased after the second vaccine injection (reaching 53±11 at day 28pv, P = 0.05, n = 3). Following challenge, HI titres were significantly higher in pigs vaccinated with the non-adjuvanted whole vaccine or adjuvanted split vaccine as compared to mock-vaccinated pigs, reaching 1,365±341, 2,730±682 and 192±64, respectively, at day 100pv (P = 0.05, n = 3).

**Table 1:** HI titres after vaccination and challenge.

	HI titres a							
	Mock		Non-adjuv. whole			Adjuv. split		
Days post-vaccination	Pig #4	Pig #8	Pig #5	Pig #6	Pig #7	Pig #1	Pig #2	Pig #3
0^b^	<8	<8	<8	<8	<8	<8	<8	<8
7	<8	<8	<8	<8	**8**	<8	<8	**8**
12	<8	<8	<8	<8	<8	**32**	<8	**16**
21^c^	<8	<8	<8	<8	<8	**64**	**128**	**128**
28	<8	<8	**64**	**32**	**64**	**64**	**128**	**128**
33	<8	<8	**64**	**32**	**32**	**64**	**128**	**128**
93^d^	<8	<8	<8	**16**	<8	<8	**32**	**128**
100	**256**	**128**	**1024**	**2048**	**1024**	**4096**	**2048**	**2048**

a : HI titres were determined using 4 HA unit per well of inactivated A/England/195/09 H1N1 influenza virus.

b : Day of vaccination (all pigs).

c : Day of boost vaccination (only pigs vaccinated with the non-adjuvanted whole vaccine).

d : Day of challenge (all pigs).

### IFNγ response was elevated only following vaccination with the adjuvanted split vaccine

**Figure 2:**
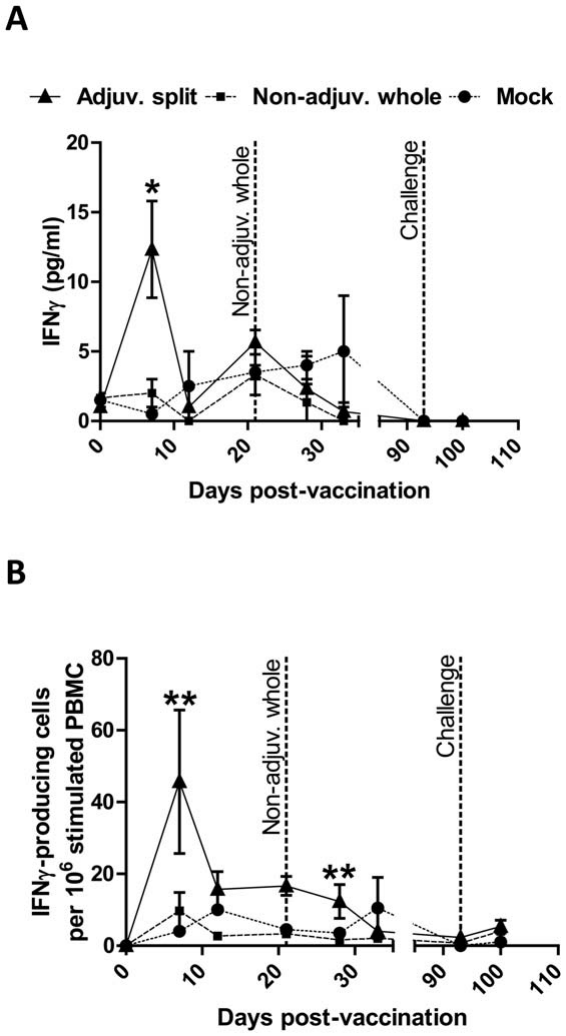
Interferon gamma response after vaccination and challenge. Pigs were vaccinated with the adjuvanted split (solid line), non-adjuvanted whole (dashed line) or mock (dotted line) vaccine. The non-adjuvanted whole vaccine was administered twice (at day 0 and day 21pv). At day 93pv, all pigs were challenged with Eng195. Blood samples were taken at various time-points post-vaccination and challenge. 2×10^5^ PBMC per well were cultured *in vitro* for 3 days in the presence of 10^5^EID_50_/ml inactivated Cal07, and interferon gamma (IFNγ) production was quantified in the supernatants by ELISA (**A**) and IFNγ-producing cells were quantified in the cellular fraction by ELISPOT (**B**). Results are expressed as the mean pg/ml (**A**) and IFNγ-producing cells per 10^6^ stimulated PBMC (**B**) ± SEM. *, P = 0.05. **, P equal to or below 0.01.

A small but significant amount of IFNγ was detected only in PBMC isolated from pigs vaccinated with the adjuvanted split vaccine at day 7pv cultured *in vitro* in the presence of inactivated Cal07 for 3 days (12±3 pg/ml, P = 0.05, n = 3) (**Figure 2A**). Indeed, no influenza-driven IFNγ was detected in the supernatants of PBMC from pigs vaccinated with the adjuvanted split vaccine isolated at any other day following the vaccination, or at any time-point from pigs vaccinated with either the non-adjuvanted whole vaccine or mock-vaccine (P>0.1, n = 3). Moreover, IFNγ production was not detected in the supernatant of PBMC isolated from any of the pigs, when cultured in the presence of mock antigen (egg allantoic fluid) or media only (data not shown).

Using *in vitro* culture conditions similar to those described above for the IFNγ ELISA, we also quantified the number of IFNγ-producing cells in pig PBMC stimulated for three days with inactivated Cal07 by ELISPOT. We detected a significant amount of influenza-driven IFNγ-producing cells in PBMC isolated from pigs vaccinated with the adjuvanted split vaccine, which peaked at day 7pv (46±20 pg/ml, adjusted P<0.01, n = 3) and subsequently remained at a low level until day 28pv (**Figure 2B**). In contrast, no influenza-driven IFNγ-producing cells were detected in PBMC from pigs vaccinated with the non-adjuvanted whole vaccine or mock-vaccine at any time-point (adjusted P = 0.99, n = 3). Similarly, IFNγ-producing cells were not detected in PBMC isolated from any of the pigs, when cultured in the presence of mock antigen (egg allantoic fluid) or media only (data not shown).

### Influenza-specific recall proliferative responses were detected in various T cell sub-populations of vaccinated pigs

**Figure 3:**
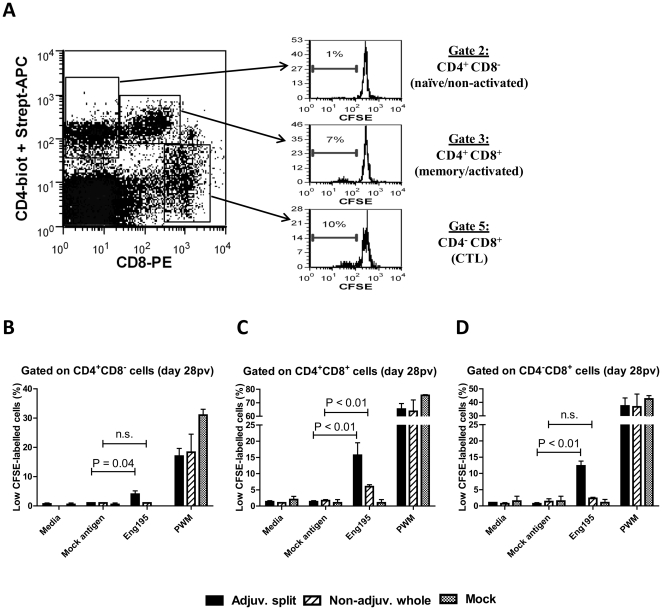
T cell sub-population proliferation monitored by CFSE labelling in response to *in vitro* re-stimulation with H1N1 influenza. CFSE-labelled PBMC were cultured *in vitro* in the presence of 10^5^EID_50_/ml inactivated Eng195, uninfected allantoic fluid (mock antigen), PWM or in media only. After 5 days, cells were stained with anti-CD4 and anti-CD8 monoclonal antibodies and analysed by flow cytometry for CFSE intensity. Three T cell subsets were identified on the basis of their CD4 and CD8 expression, and an example of the CFSE pattern at day 7pv in response to inactivated Eng195 in these various subsets in a pig vaccinated with the adjuvanted split vaccine is shown (**A**). At day 28pv, the percentage of cells proliferating (CFSE_Low_ cells) in response to media alone, mock antigen or inactivated Eng195 was determined after gating on CD4^+^CD8^−^ (**B**), CD4^+^CD8^+^ (**C**) or CD4^−^CD8^+^ (**D**) cells. Results are expressed as the mean percentage of proliferating (CFSE_Low_) cells ± SEM. n.s = Not statistically significant.

Several T cell subsets in pigs have been identified on the basis of CD4 and CD8 cell surface expression, and are generally defined as follows: naïve/non-activated CD4^+^CD8^−^ T cells, cytotoxic CD4^−^CD8^+^ T cells (CTL) and memory/activated CD4^+^CD8^+^ T cells (T_helper_) [21], [22]. Here, we assessed the level of proliferation (determined as the percentage of CFSE^low^ cells) in each of these T cell subsets within pig PBMC following their *in vitro* culture in the presence of inactivated Eng195 for 5 days (**Figure 3A**). A significant influenza-driven recall response was generated in pigs vaccinated with the adjuvanted split vaccine in the CD4^+^CD8^−^, CD4^+^CD8^+^ and CD4^−^CD8^+^ subsets (4±1%, 16±4% and 12±1% CFSE^low^ cells, respectively, P<0.05, n = 3, **Figure 3B–D**). In contrast, a significant influenza-driven recall response was only generated in the CD4^+^CD8^+^ subset (6±1% CFSE^low^ cells, P<0.01, n = 3), but not in the CD4^+^CD8^−^ and CD4^−^CD8^+^ subsets (1±0% and 2±0.3% CFSE^low^ cells, P>0.94, n = 3), in pigs vaccinated with the non-adjuvanted whole vaccine (**Figure 3B–D**). For each T cell subset, PWM was used as a positive control and induced comparable levels of proliferation in all groups of pigs, irrespective of their vaccination regime (**Figure 3B–D**). Similar results were obtained when using PBMC isolated on the day of challenge (day 93pv, data not shown). This suggests the non-adjuvanted whole vaccine only generated a T_helper_ cell response, whereas the adjuvanted split vaccine elicited a T_helper_ cell response associated with a CTL response.

### Similar levels of viral RNA were detected from the nasal cavity of pandemic- and mock-vaccinated pigs after challenge with Eng195 influenza virus

**Figure 4:**
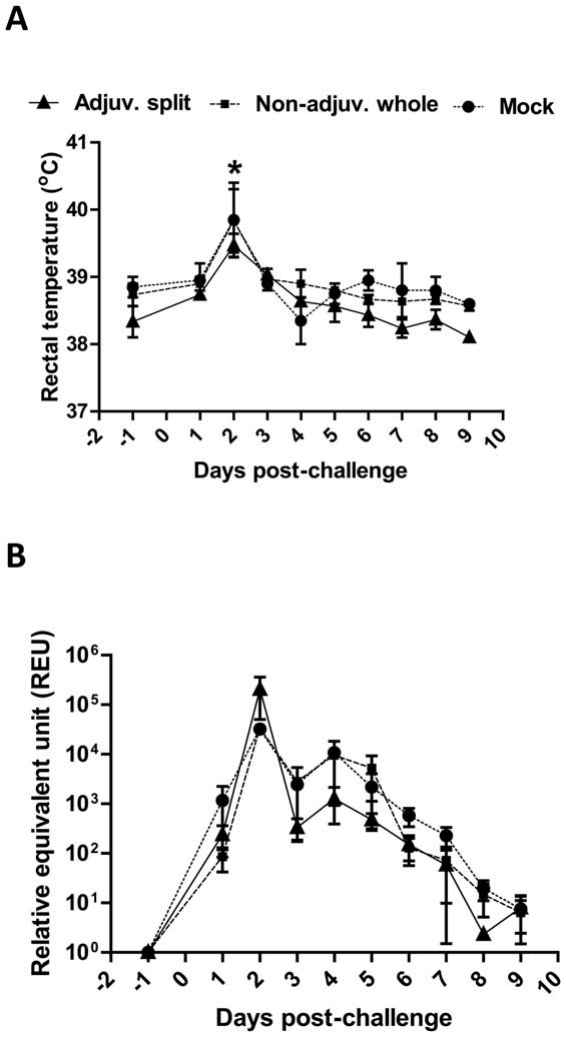
Body temperature and detection of viral RNA in the nasal cavity after infectious challenge. Pigs were vaccinated with the adjuvanted split (solid line), non-adjuvanted whole (dashed line) or mock (dotted line) vaccine, and all pigs were subsequently challenged with infectious Eng195 influenza virus. (**A**) Rectal temperature (in °C) was monitored one day prior and everyday following challenge. Results are expressed as the mean value ± SEM for each vaccination group. *, P<0.01. (**B**) Nasal swabs were collected one day prior and everyday following challenge. Shedding was measured in all samples through the detection of viral RNA by a modified influenza A M gene real-time RT-PCR (RRT-PCR) assay. By the use of a standard curve (on each test plate) generated from a dilution series of infectious Eng195 virus, the relative equivalent unit (REU) of viral RNA was determined based upon the cycle threshold (Ct) value obtained for each sample. Results are expressed as the mean REU ± SEM for each vaccination group.

Three months after the initial vaccination (day 93pv), all pigs were challenged intra-nasally with 10^6^ pfu of infectious Eng195 influenza virus. No or very mild clinical signs were observed in all pigs following this infectious challenge. Indeed, these clinical signs were: one animal in the group vaccinated with the adjuvanted split vaccine exhibited sneezing at day 2 post-challenge (pc) and one animal in the mock-vaccinated group showed some nasal discharge at days 6pc and 7pc (data not shown). Rectal temperatures were slightly elevated only at day 2pc in all pigs as follows: pigs vaccinated with the non-adjuvanted whole vaccine (from 38.7±0.2°C before challenge to 39.8±0.5°C at day 2pc, adjusted P<0.01, n = 3), pigs vaccinated with the adjuvanted split vaccine (from 38.3±0.2°C before challenge to 39.5±0.2°C at day 2pc, adjusted P<0.01, n = 3) and mock-vaccinated pigs (from 38.9±0.2°C before challenge to 39.9±0.6°C at day 2pc, adjusted P<0.01, n = 2) (**Figure 4A**).

Levels of viral RNA (indicative of shed virus) in nasal swabs taken at various time-points post-challenge were determined using a modified influenza A matrix gene RRT-PCR assay and were expressed in REU as described in materials and methods. Our results showed that similar patterns of viral RNA detected in the upper respiratory tract were observed in all pandemic- and mock-vaccinated pigs (**Figure 4B**). Indeed, all shedding curves were biphasic with a first peak of nasal shedding occurring at day 2pc (32,240±4,180 REU, 33,622±969 REU and 205,233±154,813 REU for pigs vaccinated with the mock vaccine, the non-adjuvanted whole vaccine and the adjuvanted split vaccine, respectively) and a second peak at day 4pc (10,792±1,561 REU, 9,627±8,703 REU and 1,271±882 REU for pigs vaccinated with the mock vaccine, the non-adjuvanted whole vaccine and the adjuvanted split vaccine, respectively), followed by a gradual decrease thereafter. Overall, there were no significant differences (P>0.25) in the levels of viral RNA detected between animals receiving different vaccines at any time point post challenge. Furthermore, there were no significant differences between animals in the total amount of viral RNA detected (i.e. area-under-the-curve, AUC) regardless of the vaccine received (P = 0.76).

## Discussion

In this study, we assessed the cellular and humoral immune responses generated following the vaccination of Babraham pigs with two human A(H1N1)pdm/09 influenza vaccines that were used for the national human immunisation programme in the UK.

The choice of using the pig as a model in the present study was based on the following: (i) Swine influenza viruses are enzootic in pigs, with classical swine H1N1, “avian-like” H1N1, “human-like” H1N2 and “human-” and “avian-like” H3N2 subtypes of influenza A being reported world-wide in pig populations [12]; (ii) Human and swine influenza viruses replicate to similar levels in the upper and lower respiratory tract of pigs with a similar viral shedding pattern [13], [16], [17], [23] and (iii) The pig respiratory tract exhibits a distribution of sialic acid (Sia) with α2-6 (Siaα2-6Gal, preferred receptor for human influenza virus) and α2,3 (Siaα2-3Gal, preferred receptor for avian influenza virus) linkages to galactose and a pattern of virus attachment similar to humans [13], [14]. Moreover, the Babraham inbred pigs are particularly valuable for performing immunological studies since these pigs are 85% identical as assessed by genome-wide SNP analysis (Alan Archibald, personal communication) and are matched for MHC type I and type II molecules [24]. Therefore, the genetic similarity between individual Babraham pigs also justifies the use of a lower number of animals per group (as compared to studies using outbred pigs), while allowing the assessment and dissection of the immune response in greater detail.

The influenza-specific Ab responses in the serum of vaccinated pigs were dominated by the IgG1 isotype. Such a bias towards an IgG1-dominated response has previously been demonstrated in pigs immunised with other antigens, e.g. ovalbumin or foot-and-mouth disease virus subunit [25], [26]. Pigs vaccinated with the adjuvanted split vaccine exhibited a significantly higher IgG1 response after one vaccination as compared to pigs vaccinated with the non-adjuvanted whole vaccine. However, IgG1 titres in the latter pigs were boosted after the second vaccination, and finally reached high titres (although significantly lower than those in pigs vaccinated with the adjuvanted split vaccine). Interestingly, both influenza-specific IgG1 and IgG2 titres increased following challenge with Eng195 influenza virus. This suggests that the A(H1N1)pdm/09 vaccines assessed in this study mostly generated influenza-specific IgG1 antibodies, in contrast to infection with live virus that generated a more balanced and broader immune response. Pigs vaccinated with the adjuvanted split vaccine also generated significantly higher HI titres after one vaccination, and pigs vaccinated with the non-adjuvanted whole vaccine showed a good HI response only after the boost vaccination (although lower than that observed in pigs vaccinated with the adjuvanted split vaccine). Very little data is available on the duration of neutralising antibody responses in human subjects vaccinated with these two commercial A(H1N1)pdm/09 vaccines. Recently, Madhun et al. [4] reported that health care workers vaccinated with the adjuvanted split vaccine exhibited high HI titres 21 days after the vaccination (geometric mean titre = 473.5) that were decreased three months later (geometric mean titre = 232.2). Based on this report, HI titres in human subjects were slightly higher than in pigs vaccinated with the adjuvanted split vaccine, and were slightly decreased in both species two to three months later. In another report, Waddington et al. [5] showed that HI titres in children vaccinated with the non-adjuvanted whole vaccine ranged from 60.3 to 79.6 at day 21 after the second vaccination, which is comparable to the HI values observed in pigs at day 12 after the second vaccination in our study (range 32–64). Nevertheless, duration of neutralising antibody responses could not be compared between pigs and humans from this report since the authors did not assess HI titres in these children at later time-points. HI values in children vaccinated with the adjuvanted split vaccine could not be compared since these children did not receive one but two injections of the vaccine.

In response to *in vitro* stimulation of PBMC with inactivated H1N1 influenza virus, we detected a peak of IFNγ-producing cells and IFNγ production at day 7pv, but only in pigs vaccinated with the adjuvanted split vaccine. Such an anamnestic response in the number of influenza-specific IFNγ-producing cells in the blood has similarly been detected only at day 7 after the challenge of pigs with A/Sw/Indiana/1726/88 H1N1 swine influenza virus [27]. The authors of this study further showed this response was enhanced and detectable for a longer period of time (up to day 21 post-challenge) in the spleen, tracheobronchial lymph nodes and nasal mucosa [27]. Using CFSE labelling and flow cytometry, we determined the identity of the T cell subsets with the capacity to proliferate in response to an *in vitro* re-stimulation with inactivated H1N1 influenza virus. In addition to the classical CD4^+^CD8^−^ and CD4^−^CD8^+^ T cell subsets, pig PBMCs are also known to contain a substantial number of CD4^+^CD8^+^ T lymphocytes. Importantly, these CD4^+^CD8^+^ T cells express CD8αα (not CD8αβ) molecule, are major histocompatibility complex (MHC) class II-restricted and are generally considered as memory/activated T_helper_ cells [21], [22]. Our results demonstrated an influenza-specific recall response in the CD4^+^CD8^−^ (naïve), the CD4^−^CD8^+^ (CTL) and the CD4^+^CD8^+^ (T_helper_) cells of pigs vaccinated with the adjuvanted split vaccine, but only in the CD4^+^CD8^+^ (T_helper_) cells of pigs vaccinated with the non-adjuvanted whole vaccine. It is tempting to speculate that the lack of IFNγ response observed in pigs vaccinated with the non-adjuvanted whole vaccine (even following the boost vaccination) may reflect the incapacity or at least the low efficiency of the non-adjuvanted whole vaccine to elicit an influenza-specific CTL response in these pigs. These killed vaccines are likely to stimulate CTL responses by cross-priming. It has been previously shown that some adjuvants have the ability to strongly enhance antigen cross-presentation (including that of peptide or protein antigen) [28], [29], [30]. AS03 adjuvant contained in the adjuvanted split vaccine is known to induce local inflammation and recruitment of various innate immune cells [31], and may similarly enhance antigen cross-presentation.

Despite the fact that both vaccines elicited systemic humoral responses and one of these (adjuvanted split vaccine) also elicited an enhanced cell-mediated immune response, none of the pigs were protected from a subsequent virus challenge at 3 months post-vaccination as viral RNA continued to be detected in the nasal cavity. Interestingly, although it has previously been shown that pigs with an HI titre equal to or above 20 were generally protected from a subsequent influenza challenge [32], two out of the three pigs vaccinated with the adjuvanted split vaccine still exhibited HI titres equal to or above 32 at the time of challenge but were nevertheless not protected. It is still unclear how the systemic responses generated after vaccination correlate with local mucosal responses in the respiratory tract [10] that may also contribute to reduction in virus shedding. Interestingly, a similar study was conducted in ferrets, challenged six weeks after the initial vaccination, by the pandemic influenza vaccine evaluation consortium (PIVEC) that generated results consistent with our own findings. This study showed the A(H1N1)pdm/09 vaccines reduced (adjuvanted split vaccine) or had no effect on (non-adjuvanted whole vaccine) the viral shedding from the upper respiratory tract, although the adjuvanted split vaccine did prevent viral replication in the lower respiratory tract of ferrets [33].

In conclusion, the present study provides support for the use of the pig as a valid experimental model for influenza infections in humans, including the assessment of protective efficacy of therapeutic interventions. This animal model offers improvements over that of mice and ferrets with clinical, pathological and immune responses similar to those induced in humans, and therefore has potential wide utility. The growing number of reagents to study the cell surface phenotype and function of immune cells in the pig will help dissect local and systemic responses. Moreover, the use of the inbred pig line will facilitate these investigations by allowing cell transfer studies and MHC I and II tetramer analysis.
